# Why is electroconvulsive therapy for depression more effective in older age? A causal mediation analysis

**DOI:** 10.1017/S0033291725000807

**Published:** 2025-04-10

**Authors:** Ana Jelovac, Sabine Landau, Petros Beeley, Cathal McCaffrey, Martha Finnegan, Gabriele Gusciute, Emma Whooley, Sarah McDonogh, Sarah Thompson, Anna Igoe, Kelly McDonagh, Declan M. McLoughlin

**Affiliations:** 1 Department of Psychiatry, Trinity College Dublin, St. Patrick’s University Hospital, Dublin, Ireland; 2 Department of Biostatistics and Health Informatics, King’s College London, London, UK; 3 Trinity College Institute of Neuroscience, Trinity College Dublin, Dublin, Ireland

**Keywords:** depression, depressive disorders, bipolar disorders, electroconvulsive therapy, ECT, neurostimulation, response, mediation analysis, late-life depression, psychotic depression, psychomotor disturbance, age of onset

## Abstract

**Background:**

Older people with depression exhibit better response to electroconvulsive therapy (ECT). We aimed to measure the total effect of age on ECT response and investigate whether this effect is mediated by psychotic features, psychomotor retardation, psychomotor agitation, age of onset, and episode duration.

**Methods:**

We pooled data from four prospective Irish studies where ECT was administered for a major depressive episode (unipolar or bipolar) with baseline score ≥21 on the 24-item Hamilton Depression Rating Scale (HAM-D). The primary outcome was change in HAM-D between baseline and end of treatment. The estimands were total effect of age, estimated using linear regression, and the indirect effects for each putative mediator, estimated using causal mediation analyses.

**Results:**

A total of 256 patients (mean age 57.8 [SD = 14.6], 60.2% female) were included. For every additional 10 years of age, HAM-D was estimated to decrease by a further 1.74 points over the ECT period (*p* < 0.001). Age acted on all putative mediators. Mechanistic theories, whereby a mediator drives treatment response, were confirmed for all putative mediators except age of onset. Consequently, mediation of the effect of age on change in HAM-D could be demonstrated for psychotic features, psychomotor retardation, psychomotor agitation, and episode duration but not for age of onset.

**Conclusions:**

A total of 43.1% of the effect of older age on increased ECT response was explained by the mediators. Treatment planning could be improved by preferentially offering ECT to older adults, especially if presenting with psychotic features, greater severity of psychomotor disturbance, and earlier in the episode.

## Introduction

Electroconvulsive therapy (ECT) remains the most effective somatic treatment for depression (Mutz et al., 2019). However, not all patient subpopulations benefit equally. In the meta-analytic literature, several clinical factors have been shown to be associated with an enhanced therapeutic response to ECT, including shorter episode duration (Haq et al., 2015), absence of medication resistance (Haq et al., 2015; Heijnen et al., 2010), presence of psychotic features (van Diermen et al., 2018), greater baseline depression severity (van Diermen et al., 2018), absence of personality disorder (Ferrea et al., 2024), and older age (van Diermen et al., 2018).

The association between older age and greater ECT response has long been recognized (Sackeim, 2004). Two aggregate-level meta-analyses (Haq et al., 2015; van Diermen et al., 2018) reported weak associations between study mean age and ECT response. However, several more recent large (*n* = 310–2074) multicentre studies have consistently reported a statistically significant effect of older age on ECT response in diverse geographical settings, including Australia (Sarma et al., 2024), the Netherlands (Loef et al., 2024), Norway (Sellevag et al., 2024), Scotland (Semple, Suveges, & Steele, 2024), Sweden (Brus et al., 2017), and the multinational Global ECT-MRI Research Collaboration (Blanken et al., 2023). In the USA, findings have been mixed: one large (*n* = 1698) retrospective cohort study observed no significant association between age and ECT response in a sample primarily treated with ultrabrief pulse ECT (Luccarelli, McCoy, Seiner, & Henry, 2022), a less effective ECT modality (Tor et al., 2015). These findings contrast with earlier prospective multicenter studies in the USA using brief-pulse ECT, in which older age was positively associated with ECT response (O’Connor et al., 2001; Tew et al., 1999).

The underlying mechanisms explaining why older age confers an advantage in terms of greater symptom reduction with ECT are unclear. Late-life depression is often characterized by cognitive impairment, cerebrovascular brain changes, neurodegenerative disease, and an increased burden of medical comorbidity (Taylor, 2014). Late-life depression may also present with distinctive or more severe clinical features, such as increased prevalence of psychotic features and greater severity of psychomotor disturbance (Brodaty et al., 1997). Late-life depression, however, is a heterogeneous syndrome that includes recurrent depression with an early onset, as well as late-onset depression manifesting itself for the first time late in life. Among patients with late-life depression, important etiological differences have emerged depending on age of onset, with early-onset depression characterized by greater likelihood of having personality difficulties and a positive family history of psychiatric disorder (Brodaty et al., 2001). Clinical features with increased prevalence in late-life depression, such as psychotic features, psychomotor disturbance, and later age of onset, have also been reported to contribute to a more favorable ECT response (Birkenhager, Pluijms, & Lucius, 2003; Dols et al., 2017; Heijnen et al., 2019; Hickie, Mason, Parker, & Brodaty, 1996; Petrides et al., 2001; van Diermen et al., 2020). As these factors associated with better ECT response are more commonly found in older adults, it is possible that some or all of them may explain the effect of older age on increased ECT response.

One way to unravel the roles of these clinical features is to use mediation analysis, a statistical approach used to understand the mechanisms through which an exposure (or treatment) variable affects an outcome variable. Mediators are intermediate variables that lie on the causal pathway between exposure and outcome (Imai, Keele, & Tingley, 2010). Mediation analysis quantifies the roles of mediator variables that transmit the causal effect, partitioning the *total effect* of an exposure into the *direct effect* (i.e. the effect of the exposure on the outcome not mediated by the mediators in the model) and *indirect effect* via the mediators. Two previous mediation analyses from the Netherlands have focused on the role of psychomotor disturbance and psychotic features as mediators of the relationship between age and ECT outcome (Heijnen et al., 2019; van Diermen et al., 2020). Heijnen et al. (2019) examined these putative mediators in unipolar depression treated with bilateral ECT (*n* = 96; mean age 63.9 years [SD = 12.3]). There was no significant total effect of age on ECT outcome. Age was associated with increased presence of psychotic features and psychomotor retardation but not agitation. In turn, psychotic features and psychomotor retardation were associated with better ECT outcome but agitation was not. There was a significant indirect effect of age via psychomotor retardation on ECT outcome (*p* = 0.049) but no significant indirect effect via agitation or psychotic features. In a second mediation analysis of unipolar or bipolar depression treated with right unilateral or bilateral ECT (*n* = 73; mean age 58.8 years [SD = 15.1]), van Diermen et al. (2020) proposed that the three mediators examined in the Heijnen et al. (2019) study might be indicators of a single underlying latent factor. Using a structural equation modeling approach with a latent mediator variable, they found that there was a significant total effect of age on ECT response and that this was significantly mediated by their latent factor.

However, an alternative causal model has not yet been considered in which episode duration is another potential mediator of the effect of age on ECT outcome. Older adults with severe depression are at risk of faster physical deterioration into a life-threatening state, necessitating ECT intervention sooner than younger adults. Consequently, older adults might be referred for ECT faster, resulting in a shorter episode duration, as previously reported (Birkenhager et al., 2010; Tew et al., 1999). Similarly, no previous mediation analysis has investigated whether the apparent effect of older age is explained by a later age of onset. As older patients referred for ECT also have a later age of onset (Tew et al., 1999), it is important to establish whether this difference can help explain the effect of older age on ECT outcome.

In summary, the mediation literature to date has examined only a limited number of potential mediators in small samples with inconsistent findings. The overall aim of the present study was to investigate the mechanisms by which older age at ECT initiation might lead to greater therapeutic benefit compared to younger age. Based on the above literature and clinical observations, we have identified psychotic features, psychomotor retardation, psychomotor agitation, age of onset, and episode duration as plausible potential mediators on the causal pathway between age and ECT response. Using pooled data from prospective studies carried out by our group, our specific objectives were to address the following research questions:

- *Presence of an action path (i.e. the path from exposure to mediator)*: Does age at ECT initiation affect the levels of the proposed mediators?
- *Presence of a conceptual path (i.e. the path from mediator to outcome)*: Does the level of a proposed mediator influence the degree of improvement in depressive symptoms following ECT?
- *Magnitude of the indirect effect*: To what extent can the total effect of age on improvement in depressive symptoms be explained by age-related changes in a proposed mediator?

We hypothesized that older adults would benefit more from ECT and that this effect would be explained by the above putative mediators.

## Methods

### Data sources

We pooled individual participant data from the EFFECT-Dep (Semkovska et al., 2016) and KEEP-WELL (Finnegan et al., 2019) trials and two prospective cohort studies, one of which (AMBER-Dep) has been described in detail elsewhere (Whooley et al., 2024). These studies were carried out by our group at St Patrick’s University Hospital, Dublin, Ireland, where patients were treated between May 2008 and July 2024. Design details, including study-specific recruitment periods, are presented in Supplementary Table S1. Participants were enrolled at a single site, an independent-sector inpatient psychiatric facility with a nationwide catchment area delivering approximately half of all acute ECT courses for both public and private patients in Ireland annually (Mental Health Commission, 2024).

**Figure 1.**
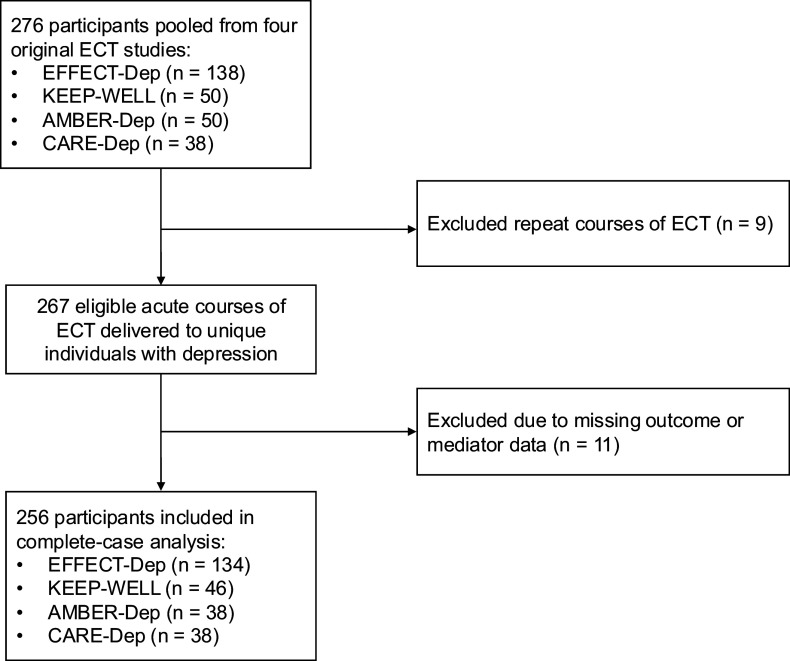
Participant flow diagram.

Eligible participants were voluntary adult (age ≥18) inpatient admissions who were referred for an acute course of ECT by their treating physician and who met *Diagnostic and Statistical Manual of Mental Disorders, Fourth Edition, Text Revision*, *Diagnostic and Statistical Manual of Mental Disorders, Fifth Edition*, or *International Statistical Classification of Diseases and Related Health Problems, 10th Revision*, criteria for a major depressive episode (unipolar or bipolar) and had a pre-ECT baseline 24-item Hamilton Depression Rating Scale (HAM-D) (Hamilton, 1967) score of ≥21. The main exclusion criteria were history of schizophrenia or schizoaffective disorder, dementia, unstable medical condition rendering the patient unfit for general anesthesia or ECT, substance-use disorder within the past 6 months, and involuntary ECT or lack of capacity to consent. Additionally, for purposes of the present mediation analysis, patients who participated in more than one of the above studies were included only once; repeat courses of ECT received by the same individual during the study period were excluded. Participant flow is shown in Figure 1.

### ECT procedures

ECT was delivered twice weekly using a MECTA spECTtrum 5000M device (MECTA Corp., Tualatin, Oregon, USA; maximum charge 1,200 millicoulomb [mC]) before September 2021 and a MECTA spECTtrum 5000Q device (MECTA Corp., Tualatin, Oregon, USA; maximum charge 1,152 mC) subsequently. Methohexital (0.5–1.0 mg/kg) was the default anesthetic, with thiopental (1.5–2.5 mg/kg) used in a minority of cases during a period of methohexital shortage. Succinylcholine (0.5–1.0 mg/kg) was used for muscle relaxation. Seizure threshold (ST) was established during the first ECT session using an empirical titration method (Semkovska et al., 2016). Participants were subsequently treated with right unilateral (d’Elia) ECT at 6 × ST (59.0% of the sample) or bitemporal ECT at 1.5 × ST (37.5% of the sample; 3.5% switched electrode placement during the course) with a brief-pulse width of 1.0 milliseconds (ms) and current amplitude of 800 mA. Stimulus dose was titrated upward during the ECT course, as required, to maintain adequate seizure duration. In rare instances of preexisting or treatment-emergent cognitive impairment, ultrabrief pulse (0.3 ms) right unilateral ECT at 6 × ST was used. Referring psychiatrists determined the duration of ECT course and choice of concomitant pharmacotherapy.

### Measures

Age at ECT initiation was the exposure of interest, treated as a continuous variable. For ease of clinical interpretation, this variable was expressed in units of decades by dividing age in years by 10.

The outcome of interest was the change in depressive symptoms measured by the HAM-D from pre-ECT baseline to the end-of-treatment visit taking place within a week of the final ECT session. HAM-D change score was derived by subtracting baseline score from the end-of-treatment score. Thus, a negative effect implies greater symptom reduction.

The following were considered as putative mediators of the exposure-outcome relationship:

- Presence of psychotic features (i.e. mood-congruent delusions and/or hallucinations) during the current depressive episode was ascertained using a combination of the Structured Clinical Interview for DSM Disorders (SCID) (First, Spitzer, Gibbon, & Williams, 1996; First, Williams, Karg, & Spitzer, 2016) at pre-ECT baseline and a review of medical records. It was coded as a binary variable (0 or 1), indicating absence or presence of psychotic features, respectively.
- Psychomotor retardation (i.e. slowing of thought and/or speech and/or decreased motor activity) was assessed using the HAM-D retardation item. It was measured on a five-point ordinal scale: 0 (normal speech and thought); 1 (slight retardation during the interview); 2 (obvious retardation during the interview); 3 (interview difficult to complete); and 4 (complete stupor).
- Psychomotor agitation (i.e. motor restlessness and/or mental tension) was measured using the HAM-D agitation item. It was evaluated on a five-point ordinal scale: 0 (none); 1 (fidgetiness); 2 (playing with hands, hair, etc.); 3 (moving about, cannot sit still); and 4 (hand wringing, nail biting, hair pulling, and biting of lips).
- Age at first onset of mood disorder was established retrospectively by reviewing medical records. This continuous variable was also divided by 10, thus expressing the effect of age of onset in decades.
- Episode duration, expressed in weeks, was a continuous variable assessed using the SCID interview at pre-ECT baseline and a review of medical records.

Depression polarity (unipolar/bipolar) and study cohort were considered as potential confounders of the exposure–outcome, exposure–mediator, and mediator–outcome relationships. Compared to unipolar depression, patients with bipolar depression may have a higher response rate following ECT and may achieve response faster (Bahji et al., 2019), although not all meta-analyses have been consistent on this matter (Haq et al., 2015). Bipolar depression can also present with more psychotic features and psychomotor disturbance and a shorter episode duration than unipolar depression (Mitchell & Malhi, 2004). Additionally, because the dataset was pooled from four individual studies that had somewhat differing inclusion criteria and ECT parameters, it is possible that study cohort affected all exposure, mediator, and outcome variables.

**Figure 2.**
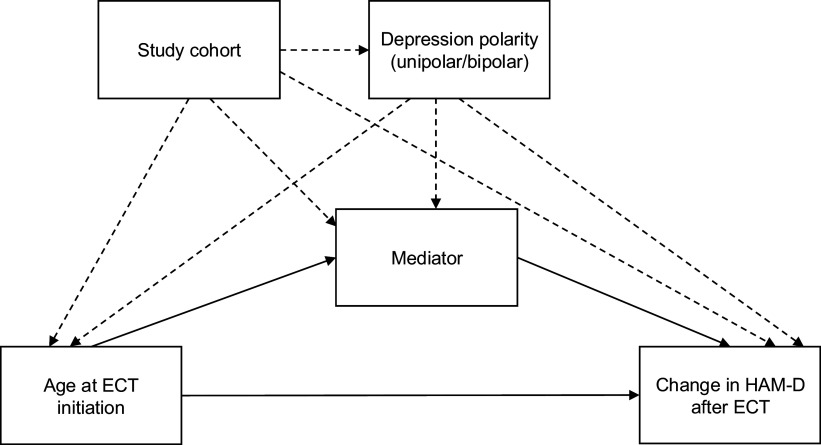
Directed acyclic graph.

Our causal model is illustrated by the directed acyclic graph (DAG) in Figure 2. The DAG visualizes the causal assumptions for our study. It shows the causal paths that we are interested in assessing (in solid lines), as well as any hypothesized confounding paths (in dashed lines). The DAG further clarifies that the direct effect of age at ECT initiation on HAM-D change across the ECT period can be estimated by conditioning on the mediator and the specified confounding variables.

### Statistical analyses

In line with our causal assumptions (Figure 2), all our analyses were conditioned on depression polarity and study cohort (three dummy variables) by including these variables as additional covariates in respective analysis models. The total effect of age at ECT initiation on depression response was estimated by regressing the depression change score on age, as well as on confounding variables.

To address our mediation-specific research questions, we fitted a single-mediator model for each potential mediator. This approach estimates three estimands that map onto the action effect, conceptual effect, and indirect effect of the respective mediator. Specifically, causal mediation analysis based on parametric modeling was used, which requires fitting (generalized) regression models for both the mediator and outcome variables, followed by estimating relevant mediation estimands. We assumed the following parametric models for the mediators: the continuous variable, age of onset/10, followed a normal distribution and included age and confounding variables as explanatory variables. This model parameterized the action effect by the regression coefficient of age/10, expressed as the expected change in age of onset per additional year of age. The positively skewed mediator, episode duration, was natural logarithm (ln)-transformed to achieve a symmetric distribution and modeled in a similar fashion to age of onset. Binary or ordinal mediators, such as psychotic features, psychomotor retardation, and agitation, were modeled using ordinal probit regression, which assumes an underlying standard-normal latent ‘liability variable’ that is converted into an ordinal variable as certain thresholds are reached. Explanatory variables for these mediator models were the same as those used for the age of onset mediator. In probit models, the action effect of age on the mediators is expressed by the expected change on a standardized liability scale (SD = 1) per 10 additional years of age. The outcome variable in the single-mediator models was the change in HAM-D score over the ECT period. This was assumed to follow a normal distribution and included age, confounders, and the putative mediator under investigation as explanatory variables (Figure 2). Thus, a different regression model was fitted to HAM-D change for each putative mediator, with two effects estimated for each mediator: the conceptual effect, measured by the expected change in HAM-D score per unit change in the putative mediator, and the (natural) direct effect of age, measured by the expected change in HAM-D score per 10 additional years of age, conditional on the value of the mediator. We assumed that action, conceptual, and direct effects did not vary with study cohort. We also assumed no interactions between mediators and age in their effects on HAM-D change and, therefore, partitioned the total effect of age as the sum of the natural direct effect and the natural indirect effect of age.

Estimates of relevant mediation estimands for each single-mediator model were obtained by fitting respective parametric models using maximum-likelihood estimation, yielding estimates of action and conceptual effects. The natural indirect effect of age was then estimated by the difference between the estimated total effect of age and the estimated direct effect of age, using the so-called difference approach to mediation (Jiang & VanderWeele, 2015). Nonparametric bootstrapping with 2000 replications was used to generate 95% percentile confidence intervals, rather than using model-based inferences to take account of nonsymmetric sampling distributions, particularly of the indirect effect estimates.

Finally, to determine the extent to which the total effect of age could be explained by the mediators jointly, we fitted a multiple-mediator model incorporating variables that demonstrated significant mediation effects individually. All significant mediators were included in the model for HAM-D change, providing an estimate of the residual direct (non-mediated) effect of age and the indirect effect, again derived by the difference method. All statistical analyses were performed using Stata 18 (StataCorp, College Station, Texas, USA).

## Results

### Sample characteristics

The initial combined study sample included 267 patients receiving ECT for depression. Eleven patients with missing values for either the HAM-D outcome (missing for nine patients) or one or more putative mediators (missing for two patients) were excluded (Figure 1). Table 1 presents the sociodemographic and clinical characteristics of the complete-case sample (*n* = 256), indicating that the study participants had a mean age of 57.8 years, were of white ethnic background (100%), majority female (60.2%) and majority married/cohabiting (59.8%). The rising mean age between earliest (EFFECT-Dep) and most recent (CARE-Dep) datasets is consistent with the rising mean age of ECT referrals in the population of all ECT referrals in Ireland from a mean of 57 in 2010 to 63 in 2022 (Mental Health Commission, 2024). ECT treatment parameters have also been optimized over time, with all patients in the most recent dataset receiving high-dose right unilateral brief-pulse ECT, which is equivalent in efficacy to bitemporal ECT but results in significantly less retrograde amnesia (Kolshus, Jelovac, & McLoughlin, 2017). The majority of participants were receiving concomitant antidepressants and one or more augmentation strategies, as would be expected in a patient population with moderate-to-severe depression.

**Table 1.** Sociodemographic and clinical characteristics by study (*n* = 256)

Variable	Total sample (*n* = 256)	EFFECT-Dep(*n* = 134)	KEEP-WELL(*n* = 46)	AMBER-Dep (*n* = 38)	CARE-Dep(*n* = 38)
Age (years), mean (SD)	57.8 (14.6)	56.9 (14.5)	59.5 (15.7)	53.3 (13.1)	63.2 (13.8)
Female, *n* (%)	154 (60.2%)	84 (62.7%)	26 (56.5%)	24 (63.2%)	20 (52.6%)
White, *n* (%)	256 (100%)	134 (100%)	46 (100%)	38 (100%)	38 (100%)
Employment status, *n* (%)					
Employed or student	93 (36.3%)	55 (41.0%)	11 (23.9%)	17 (44.7%)	10 (26.3%)
Unemployed	58 (22.7%)	32 (23.9%)	11 (23.9%)	10 (26.3%)	5 (13.2%)
Retired	105 (41.0%)	47 (35.1%)	24 (52.2%)	11 (29.0%)	23 (60.5%)
Marital status, *n* (%)					
Married or cohabiting	153 (59.8%)	76 (56.7%)	33 (71.7%)	19 (50.0%)	25 (65.8%)
Single	61 (23.8%)	33 (24.6%)	5 (10.9%)	14 (36.8%)	9 (23.7%)
Separated or divorced	21 (8.2%)	13 (9.7%)	5 (10.9%)	2 (5.3%)	1 (2.6%)
Widowed	21 (8.2%)	12 (9.0%)	3 (6.5%)	3 (7.9%)	3 (7.9%)
Baseline 24-item Hamilton Depression Rating Scale, mean (SD)	29.0 (6.1)	29.9 (6.3)	30.0 (7.4)	26.3 (3.7)	27.0 (4.4)
Age at first onset of mood disorder (years), mean (SD)	40.8 (17.9)	41.0 (17.0)	40.9 (18.6)	32.6 (15.8)	48.0 (19.2)
Number of previous episodes, median (min, max)	3 (0, 120)	4 (0, 23)	3 (0, 10)	3 (0, 20)	2.5 (0, 120)
Duration of index episode in weeks, median (min, max)	22 (2, 1040)	19.5 (2, 520)	23 (3, 187)	39 (7, 1040)	22 (2.5, 156)
Bipolar depression, *n* (%)	38 (14.8%)	32 (23.9%)	0 (0.0%)	4 (10.5%)	2 (5.3%)
Psychotic symptoms, *n* (%)	38 (14.8%)	28 (20.9%)	5 (10.9%)	4 (10.5%)	1 (2.6%)
Psychomotor retardation baseline severity, *n* (%)					
0	146 (57.0%)	74 (55.2%)	32 (69.6%)	22 (57.9%)	18 (47.4%)
1	73 (28.5%)	36 (26.9%)	7 (15.2%)	12 (31.6%)	18 (47.4%)
2	32 (12.5%)	21 (15.7%)	5 (10.9%)	4 (10.5%)	2 (5.3%)
3	5 (2.0%)	3 (2.2%)	2 (4.4%)	0 (0.0%)	0 (0.0%)
Psychomotor agitation baseline severity, *n* (%)					
0	171 (66.8%)	84 (62.7%)	29 (63.0%)	26 (68.4%)	32 (84.2%)
1	50 (19.5%)	28 (20.9%)	8 (17.4%)	9 (23.7%)	5 (13.2%)
2	29 (11.3%)	19 (14.2%)	7 (15.2%)	2 (5.3%)	1 (2.6%)
3	6 (2.3%)	3 (2.2%)	2 (4.4%)^a^	1 (2.6%)	0 (0.0%)
History of previous ECT, *n* (%)	93 (36.3%)	52 (38.8%)	17 (37.0%)	11 (29.0%)	13 (34.2%)
Number of ECT sessions, mean (SD)	8.6 (2.7)	8.0 (2.6)	9.3 (2.8)	9.0 (2.9)	9.5 (2.2)
Electrode placement, *n* (%)					
Right unilateral	151 (59.0%)	65 (48.5%)	22 (47.8%)	26 (68.4%)	38 (100.0%)
Bitemporal	96 (37.5%)	69 (51.5%)	19 (41.3%)	8 (21.1%)	0 (0.0%)
Switch from unilateral to bitemporal	3 (1.2%)	0 (0.0%)	3 (6.5%)	0 (0.0%)	0 (0.0%)
Switch from bitemporal to unilateral	6 (2.3%)	0 (0.0%)	2 (4.4%)	4 (10.5%)	0 (0.0%)
Pulse width, *n* (%)					
Brief (1.0 ms)	253 (98.8%)	134 (100.0%)	46 (100.0%)	37 (97.4%)	36 (94.7%)
Ultrabrief (0.3 ms)	1 (0.4%)	0 (0.0%)	0 (0.0%)	0 (0.0%)	1 (2.6%)
Switch from brief to ultrabrief	2 (0.8%)	0 (0.0%)	0 (0.0%)	1 (2.6%)	1 (2.6%)
Baseline psychotropic medications, *n* (%)					
Antidepressants	241 (94.1%)	129 (96.3%)	43 (93.5%)	31 (81.6%)	38 (100.0%)
Antipsychotics	193 (75.4%)	100 (74.6%)	33 (71.7%)	31 (81.6%)	29 (76.3%)
Lithium	106 (41.4%)	53 (39.6%)	19 (41.3%)	17 (44.7%)	17 (44.7%)
Anticonvulsants	65 (25.4%)	42 (31.3%)	8 (17.4%)	8 (21.1%)	7 (18.4%)
Benzodiazepines	140 (54.7%)	78 (58.2%)	21 (45.7%)	27 (71.1%)	14 (36.8%)
Z-hypnotics	118 (46.1%)	72 (53.7%)	16 (34.8%)	14 (36.8%)	16 (42.1%)

a One patient in category 4 merged with category 3 to avoid perfect prediction issues in statistical analyses.

A total of 55.9% of the sample achieved a final HAM-D score of ≤10, indicating remission, with 65.6% achieving a reduction in HAM-D score of ≥50% from baseline, indicating clinical response.

### Does age at ECT initiation affect the treatment response?

**Figure 3.**
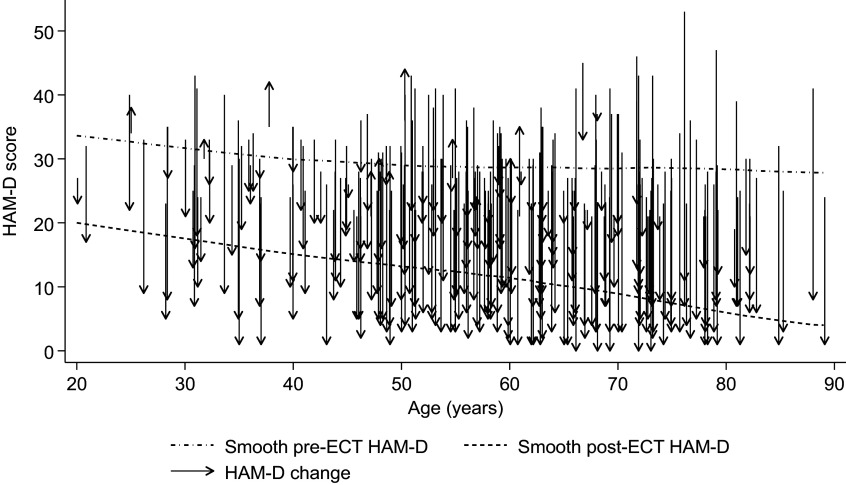
Change in HAM-D scores following ECT across the age range.

Figure 3 illustrates the observed changes in HAM-D scores following ECT. A smooth lowess curve of pre-ECT HAM-D scores against age indicates that baseline HAM-D scores were relatively constant across age-groups. Post-ECT HAM-D scores were generally lower than baseline scores (mean reduction = 17.4 points, *t* = -28.0, df = 255, *p* < 0.001; see Supplementary Table S2 for HAM-D scores in each of the four studies). The smooth lowess curve also suggests a downward trend in post-ECT HAM-D scores with increasing age. This trend was confirmed by formal analysis: after adjusting for study and polarity, each additional 10 years of age at ECT initiation was associated with a 1.74-point greater reduction in HAM-D score over the ECT period (95% CI: −2.56 to −0.97, *t* = −4.08, df = 250, *p* < 0.001; standardized regression coefficient = 0.26).

### Does age at ECT initiation affect the levels of the proposed mediators?

Table 2 summarizes the action effects for each of the five putative mediators. All five mediators were significantly influenced by age at ECT initiation: as expected, for each additional 10 years of age at ECT initiation, the mean age at depression onset was also later, increasing by 7.7 years. Additionally, older age at ECT initiation led to a shorter episode duration: the estimated decrease in the duration of the episode for a 10-year increase in age was 8.0%. Older patients also had higher liabilities for presenting with psychotic features and psychomotor retardation and agitation, with estimated increases of 0.17, 0.14, and 0.15 standard deviations (SD), respectively, per 10 additional years of age. Thus, age at ECT initiation acted on all the hypothesized mediator variables.

**Table 2.** Results of mediation analyses for the effect of age on change in HAM-D after ECT

Mediator	Action effect Effect of age (years/10) on mediator	Conceptual effect Effect of mediator on change in HAM-D	Indirect effect Effect of age (years/10) on change in HAM-D
Psychotic features (presence/absence)	**0.17** (0.022, 0.353)	**−5.67** (**−**9.26, **−**2.42)	**−0.20** (**−**0.47, **−**0.018)
Retardation (Likert 0–3)	**0.14** (0.044, 0.261)^a^	**−2.02** (**−**3.59, **−**0.35)	**−0.19** (**−**0.47, **−**0.022)
Agitation (Likert 0–3)	**0.15** (0.038, 0.272)^b^	**−3.23** (**−**4.75, **−**1.69)	**−0.28** (**−**0.56, **−**0.064)
Age of onset (years/10)	**0.77** (0.65, 0.88)	–0.59 (**−**1.57, 0.38)	**−**0.45 (**−**1.24, 0.28)
Episode duration in weeks (ln-transformed)	**−0.083** (**−**0.159, **−**0.007)	**1.70** (0.60, 2.78)	**−0.14** (**−**0.329, **−**0.002)

Estimated effect sizes and 95% CIs for action effects, conceptual effects, and natural indirect effects (*n* = 256). All estimates are adjusted for study and depression polarity (unipolar/bipolar). Inferences by percentile bootstrap (2000 replications). Statistically significant (5% test level) effect size estimates are shown in bold.

a Sixteen bootstrap samples excluded due to perfect prediction (negligible).

b Four bootstrap samples excluded due to perfect prediction (negligible).

### Does the level of a proposed mediator influence the degree of improvement in depressive symptoms following ECT?

Table 2 also summarizes the conceptual effects for each of the five putative mediators. Age of onset did not significantly affect HAM-D change (*p* > 0.05). By contrast, mechanistic relationships were observed between each of the remaining four mediators and HAM-D change (*p* < 0.05). Specifically, longer episode duration resulted in a poorer ECT response; with each unit increase in ln-duration of episode (range: 0.69–6.95), HAM-D change was reduced by 1.70 points. We also estimated an additional improvement in HAM-D of 5.67 points for patients with psychotic features compared to those without, and an additional improvement of 2.02 and 3.23 for every additional point on the retardation and agitation scales, respectively. Thus, mechanisms involving all the putative mediators, except for age of onset, were empirically confirmed by our study.

### To what extent can the total effect of age on improvement in depressive symptoms be explained by age-related changes in a proposed mediator?

Finally, Table 2 summarizes the (natural) indirect effects of each putative mediator when analyzed individually within a single-mediator model. The indirect effect of age at ECT initiation quantifies how much of the total effect of age on HAM-D change (1.74 points per 10 years of age) can be explained by age at ECT initiation changing the mediator’s value, which subsequently affects the outcome via the conceptual path. Confidence intervals shown in Table 2 indicate that all mediators, except age of onset, significantly explained some of the total effect (*p* < 0.05). Among these, severity of psychomotor agitation accounted for the largest proportion of the total effect (indirect effect estimate = −0.28, explaining 16.1% of the total effect), followed by presence of psychotic features (indirect effect = −0.20 or 11.5%), severity of psychomotor retardation (indirect effect = −0.19 or 10.9%), and episode duration (indirect effect = −0.14 or 8.0%).

### Multiple-mediator analysis

In single-mediator models, presence of psychotic features, retardation, agitation severity, and episode duration were confirmed as mediators. However, a positive correlation was observed between presence of psychotic features and severity of retardation symptoms, partialing out the effects of age (*r* = 0.25), suggesting that some indirect effects could reflect a common underlying symptom dimension. To disentangle this, we proceeded to estimate the (natural) direct effect of age at ECT initiation on ECT response in a multiple-mediator model incorporating these four mediators, resulting in an estimate of a 0.99-point reduction per 10 additional years of age (95% CI: −1.75 to −0.27) and a resulting combined (natural) indirect effect of −0.75 points (95% CI: −1.18 to −0.39). These findings demonstrate that the four mediators contributed independently to explaining the total age effect, together accounting for 43.1% of the total effect. However, the substantial remaining direct effect (56.9%) suggests additional, unmodeled pathways by which older age at ECT initiation enhances ECT response.

## Discussion

Our findings reaffirmed the clinically meaningful effect of older age on ECT outcome and quantify the role of four clinical features, namely psychotic features, psychomotor retardation and agitation, and episode duration, as mediators of this relationship. Together, these mediators accounted for nearly half of the total effect of age on ECT outcome. Identifying clinical features driving ECT outcome offers a notable advantage over more costly or less accessible diagnostics such as neuroimaging or genotyping, because clinical features are readily clinically observable or assessable.

Our findings are broadly consistent with the general conclusions of previous mediation analyses. Heijnen et al. (2019) did not detect a total effect of age on ECT outcome, nor did they find a statistically significant indirect effect via psychomotor agitation or psychotic features. However, unlike in our study, Heijnen et al. (2019) treated age as an ordinal exposure, with participants divided into three broad age categories, while all three mediators were dichotomized into present or absent categories. The absence of any evidence for some of the expected effects may be due to low power of analyses with binary mediators and a limited sample size. Our results are consistent with the van Diermen et al. (2020) mediation analysis, in which (standardized) total effect of age on depression improvement following ECT was 0.39 compared with 0.26 in our study. It should be noted that both previous mediation analyses included an unusually high proportion of patients with psychotic features: 59% in Heijnen et al. (2019) and 45% in van Diermen et al. (2020).

Our findings question the conventional wisdom codified in current treatment algorithms for depression where ECT is typically relegated to ‘third-line’ status (Simon, Moise, & Mohr, 2024), to be considered only after all other approaches have failed. Given that a longer episode duration portends a worse ECT outcome (Haq et al., 2015), and considering the diminished efficacy and tolerability of conventional monoaminergic antidepressant treatments for late-life depression (Tham et al., 2016), our findings imply that ECT could be preferentially offered to older patients, especially those with psychotic features and psychomotor disturbance, earlier in their treatment course. On the other hand, as ECT involves exposure to risks of general anesthesia and retrograde amnesia, fine-tuning the delivery of this treatment is crucial, ensuring it is prioritized for those most likely to benefit. This will help to avoid exposing to risks patients who are unlikely to benefit substantially from ECT, such as younger adults without any biological signs of depression and/or prolonged or chronic depressive symptoms.

The strengths of the present study include its hypothesis-driven approach to model building, a relatively large sample size, and the use of robust statistical methods for handling binary and ordinal mediators. However, certain limitations should be acknowledged. First, the single-center design may limit the generalizability of findings to other populations. Second, the prospective nature of the studies from which the datasets were sourced prevented the inclusion of the most acutely ill ECT patients. As such individuals were unable to participate due to lacking capacity to consent, this precluded inclusion of the most severe manifestations of psychomotor retardation (i.e. catatonia) and agitation. Third, the high psychotropic medication burden, particularly benzodiazepines and anticonvulsant mood stabilizers, reflects routine clinical practice but may have influenced treatment outcome by affecting the seizure threshold. Fourth, psychomotor agitation and retardation, two of our mediators, were assessed using single HAM-D items. Single-item measurements of these constructs may be less reliable than assessments based on multi-item scales. Lastly, the four clinical factors identified as significant mediators accounted for 43.1% of the total effect of age on symptom reduction following ECT, which suggests the presence of additional causal mechanism(s) warranting investigation in future studies. These mechanisms may include suicidality (Blanken et al., 2025; Sienaert et al., 2022), body mass index (Nakajima et al., 2022), and comorbid personality disorders (Ferrea et al., 2024).

## Conclusion

Our findings support the notion that older adults with psychotic features, psychomotor disturbance, and shorter episode duration benefit more from ECT and highlight the importance of prioritizing ECT use in such patients. It is hoped that future research will illuminate other currently unknown causal pathways between older age and ECT response, further optimizing treatment delivery for this vulnerable patient population.

## Supporting information

Jelovac et al. supplementary material Jelovac et al. supplementary material

## Supplementary Material

The supplementary material for this article can be found at http://doi.org/10.1017/S0033291725000807.
