# Supplementary material for: Why is electroconvulsive therapy for depression more effective in older age? A causal mediation analysis
Source: Psychol Med. 2025 Apr 10;55:e110. doi: 10.1017/S0033291725000807 (PMC12094667; doi:10.1017/S0033291725000807)
Supplement: Jelovac et al. supplementary material [file S0033291725000807sup001.docx]

**Supplementary Material**

**Why is electroconvulsive therapy for depression more effective in older age? A causal mediation analysis**

The EFFECT-Dep Trial (Semkovska et al., 2016) was a randomised controlled non-inferiority trial of high-dose right unilateral versus bitemporal ECT. The KEEP-WELL Trial (Finnegan et al., 2019) was a pilot trial of intravenous ketamine versus midazolam for post-ECT relapse prevention; the data used in the present mediation analysis were from the non-interventional phase of this study. The aims of the AMBER-Dep (Whooley et al., 2024) and CARE-Dep (unpublished) prospective cohort studies were to examine retrograde amnesia and clinical factors associated with ECT response, respectively. Details of all four studies are presented in Table S1 below.

**Table S1**. Characteristics of ECT studies conducted at St Patrick’s University Hospital, Ireland since 2008

| **Study** | **Recruitment period** | **Study design** | **Inclusion criteria** | **Exclusion criteria** |
| --- | --- | --- | --- | --- |
| EFFECT-Dep (Semkovska et al., 2016) | 2008-2012 | Randomised controlled trial | - Age ≥18 years - Met DSM-IV diagnostic criteria for major depressive episode (unipolar or bipolar) confirmed using the Structured Clinical Interview for DSM Disorders (SCID) - Scored ≥21 on the 24-item Hamilton Depression Rating Scale (HAM-D) at pre-ECT baseline | - Medical conditions rendering patients unﬁt for general anaesthesia or ECT - ECT in the previous six months - History of schizophrenia, schizoaffective disorder or dementia - Alcohol/substance abuse in the previous six months - Involuntary status - Inability or refusal to consent |
| KEEP-WELL (Finnegan et al., 2019) | 2015-2017 | Observational run-in period during ECT course (prospective cohort study); randomised controlled trial during post-ECT relapse prevention phase | - Age ≥18 years - Met DSM-IV diagnostic criteria for unipolar major depressive episode confirmed using the SCID - Scored ≥21 on the 24-item HAM-D at pre-ECT baseline | - Allergy/sensitivity to study medications or their ingredients. - Subjects who have participated in another study and received any other investigational agent within 6 months. - Any condition rendering patient medically unfit for ECT, general anaesthesia, ketamine or midazolam - assessed by physical examination, routine haematology and biochemistry investigations prior to enrolment. - Medications that may significantly alter the pharmacokinetics of ketamine (e.g. ketoconazole, clarithromycin) are contraindicated during the trial, and participants taking any of these medications at screening will be excluded from the trial. - Subjects who have a history of drug or alcohol use that, in the opinion of the investigator, would interfere with adherence to study requirements. - Known history of, or documented positive hepatitis B or C or HIV infection, advanced malignancy or terminal illness. - Scheduled for non-trial procedures requiring general anaesthesia during the study. - Active suicidal intent. - Dementia, intellectual disability or a score of <24 on the standardised Mini Mental State Examination. - Lifetime history of bipolar affective disorder. - Current post-traumatic stress disorder. - Other Axis I diagnosis (DSM-IV). - ECT in the six months prior to recruitment. - 14. Currently a prisoner or residing in a nursing home. |
| AMBER-Dep (Whooley et al., 2024) | 2017-2019 | Prospective cohort study | - Age ≥18 years - Met ICD-10 clinical criteria for a depressive episode (unipolar or bipolar) - Scored ≥21 on the 24-item HAM-D at pre-ECT baseline | - Conditions rendering patients unﬁt for general anaesthesia or ECT - ECT in the previous six months - History of schizophrenia, schizoaffective disorder or neurological disorder - Alcohol/substance abuse in the previous six months - Involuntary status - Inability or refusal to consent |
| CARE-Dep (unpublished) | 2023-2024 | Prospective cohort study | - Age ≥18 years - Met DSM-5 diagnostic criteria for a major depressive episode (unipolar or bipolar) confirmed using the SCID - Scored ≥21 on the 24-item HAM-D at pre-ECT baseline | - Lifetime diagnosis of schizoaffective disorder or schizophrenia - Active psychiatric comorbidity in the past six months (except anxiety disorders if deemed secondary to depression) - Substance use disorder in the past six months - Acute course of ECT in the past three months (maintenance ECT in the past three months is permitted) - Neurological or active/unstable major medical condition that may affect memory function - Lack of capacity to consent to research or involuntary status |

**Table S2**. Summaries of HAM-D scores and change in HAM-D scores by ECT study

| **Variable** | **Total sample (*n* = 256)** | **EFFECT-Dep (*n* = 134)** | **KEEP-WELL (*n* = 46)** | **AMBER-Dep (*n* = 38)** | **CARE-Dep (*n* = 38)** |
| --- | --- | --- | --- | --- | --- |
| Pre-ECT HAM-D, mean (SD) | 29.0 (6.1) | 29.9 (6.3) | 30.0 (7.4) | 26.3 (3.7) | 27.0 (4.4) |
| Post-ECT HAM-D, mean (SD) | 11.6 (8.9) | 11.8 (8.4) | 13.0 (10.9) | 11.0 (7.4) | 9.5 (9.3) |
| Change in HAM-D, mean (SD) | -17.4 (10.0) | -18.2 (10.3) | -16.9 (12.5) | -15.3 (6.6) | -17.5 (7.6) |

**References**

Finnegan, M., Galligan, T., Ryan, K., Shanahan, E., Harkin, A., Daly, L., & McLoughlin, D. M. (2019). Ketamine versus midazolam for depression relapse prevention following successful electroconvulsive therapy: a randomized controlled pilot trial. *J ECT*, *35*(2), 115-121. <https://doi.org/10.1097/YCT.0000000000000560>

Semkovska, M., Landau, S., Dunne, R., Kolshus, E., Kavanagh, A., Jelovac, A., Noone, M., Carton, M., Lambe, S., McHugh, C., & McLoughlin, D. M. (2016). Bitemporal versus high-dose unilateral twice-weekly electroconvulsive therapy for depression (EFFECT-Dep): a pragmatic, randomized, non-inferiority trial. *Am J Psychiatry*, *173*(4), 408-417. <https://doi.org/10.1176/appi.ajp.2015.15030372>

Whooley, E., Gusciute, G., Kavanagh, K., McDonagh, K., McCaffrey, C., Doody, E., Jelovac, A., & McLoughlin, D. M. (2024). Reliable change indices and minimum detectable change for the Montreal Cognitive Assessment in electroconvulsive therapy for depression. *J ECT*. <https://doi.org/10.1097/YCT.0000000000001043>
